# Structural insights into inhibition of PRRSV Nsp4 revealed by structure-based virtual screening, molecular dynamics, and MM-PBSA studies

**DOI:** 10.1186/s13036-022-00284-x

**Published:** 2022-02-22

**Authors:** Rajesh Kumar Pathak, Young-Jun Seo, Jun-Mo Kim

**Affiliations:** Department of Animal Science and Technology, Chung-Ang University, Anseong-si, Gyeonggi-do 17546 Republic of Korea

**Keywords:** PRRSV, Swine, Nsp4, Molecular dynamics, Protein–ligand interaction

## Abstract

**Background:**

Porcine reproductive and respiratory syndrome respiratory sickness in weaned and growing pigs, as well as sow reproductive failure, and its infection is regarded as one of the most serious swine illnesses worldwide. Given the current lack of an effective treatment, in this study, we identified natural compounds capable of inhibiting non-structural protein 4 (Nsp4) of the virus, which is involved in their replication and pathogenesis.

**Results:**

We screened natural compounds (*n* = 97,999) obtained from the ZINC database against Nsp4 and selected the top 10 compounds for analysing protein–ligand interactions and physicochemical properties. The five compounds demonstrating strong binding affinity were then subjected to molecular dynamics simulations (100 ns) and binding free energy calculations. Based on analysis, we identified four possible lead compounds that represent potentially effective drug-like inhibitors.

**Conclusions:**

These methods identified that these natural compounds are capable of inhibiting Nsp4 and possibly effective as antiviral therapeutics against PRRSV.

## Introduction

Porcine reproductive and respiratory syndrome virus (PRRSV) infection is an economically important disease in swine and accountable for significant losses to the pork industry worldwide [1]. PRRSV is an enveloped, single-stranded RNA virus of the genus *Arterivirus* [1–4] that causes respiratory disease and is responsible for severe reproductive failure in pigs [4]. Generally, the disease is further complicated by secondary infection and results in a high mortality rate [1]. The available treatments, including vaccines, poorly control the disease, owing to the high genetic and antigenic heterogeneity of the virus [5]. Antivirals might be useful in controlling and managing PRRSV, and recent studies have reported the ability of herbal extracts to inhibit PRRSV infection [6–8]. Many compounds derived from natural sources such as plants have shown inhibitory activity against viruses and a wide variety of pathogens [9, 10]. Several natural compounds have demonstrated antiviral activity (for instance, immense potential for inhibiting viral replication) and drug-like activity [10]. The identification of potent inhibitors of PRRSV from natural sources is challenging, as it requires compound extraction and evaluation of antiviral activity, both of which require funds and specific experimental equipment. Given the difficulty in screening large numbers of natural compounds, chemo-informatics approaches are useful for identifying lead compounds from databases by targeting essential viral proteins [11, 12].

Open reading frame (ORF)1a and ORF1b comprise ~ 80% of the PRRSV genome and respectively encode pp1a and pp1b polyproteins that are cleaved by viral proteases into non-structural proteins [13]. Papain-like proteases (PL1pro and PL2pro) and a 3C-like serine protease [3CLSP; non-structural protein 4 (Nsp4)] are the viral proteases involved in polyprotein cleavage and required for *Arterivirus* replication. Additionally, the reported involvement of Nsp4 in interferon inhibition is linked with PRRSV pathogenesis, suggesting it as a promising molecular target for novel therapeutics [14–16].

The goal of this study was to identify natural compounds capable of serving as novel inhibitors of PRRSV replication via their targeting of Nsp4. Specifically, we aimed to identify natural compounds through structure-based virtual screening, analyse their physicochemical properties, perform molecular dynamics (MD) simulations, and determine the binding affinities of the potential inhibitors using molecular mechanics Poisson–Boltzmann surface area (MM-PBSA) methods.

## Materials and methods

### Retrieval and preparation of ligand structures

We retrieved 97,999 compounds from a subset of the ZINC database housing natural compounds [17]. All natural compounds were downloaded in the structure-data file format, and these files were subsequently converted to AutoDock PDBQT [Protein Data Bank (PDB), partial charge (Q), and atom type (T)] files using OpenBabel software (https://openbabel.org/wiki/Main_Page). These files were then used for interaction studies with target protein through structure-based virtual screening and molecular docking using AutoDock vina [18–20].

### Retrieval and preparation of the target protein structure

The crystal structure of PRRSV Nsp4 (PDB ID: 5Y4L) was retrieved from RCSB-PDB and visualised and analysed using UCSF Chimera-1.15 software [21, 22]. An AutoDock tool was used for the addition of partial atomic charges (Kollman charge), hydrogen atoms, generation of gridbox, and preparation of the Nsp4 structure. The grid box was generated with centre (X = − 4.034, Y = 6.285, Z = 17.57) and size (X = 46, Y = 44, Z = 42) coordinates that were defined in a configuration file (exhaustiveness and energy ranges: 8 and 4, respectively). The prepared structure was saved in the PDBQT file format for molecular docking [23].

### Structure-based virtual screening

Virtual screening is a computational method widely used for the identification of lead molecules by docking large numbers of compounds with a molecular target of interest to allow evaluation of the binding free energy of the docked/screened compounds using drug discovery programs [24]. AutoDock vina is a molecular docking and virtual screening program that determines the preferred relative orientation of a ligand during docking or interaction with a molecular target and provides a stable protein–ligand complex structure that exhibits a minimum binding energy [20]. Here, we used AutoDock vina to screen the retrieved natural compounds against PRRSV Nsp4 and generated protein–ligand complexes of the top 10 screened compounds using PyMol (https://pymol.org/2/). Two-dimensional (2D) models of the complexes were visualised using Discovery Studio Visualizer (https://discover.3ds.com/discovery-studio-visualizer-download) to determine the amino acid residues involved in the interactions [25].

### Drug-likeness analysis

A total of seven principal descriptors were included to evaluate the drug-likeness of the top 10 screened natural compounds. These included molecular weight (MW), logP value, status as a hydrogen-bond donor (HBD), and acceptor (HBA), polar surface area (2D; PSA), polarizability (P), and van der Waals surface area (VWSA). MW, logP, HBD, and HBA were obtained from the ZINC database [17], whereas PSA, P, and VWSA were calculated using the MarvinSketch software (https://chemaxon.com/products/marvin).

### MD simulation

The MD simulations were performed using GROMACS (v.2018.1; http://www.gromacs.org/) for stability predictions of the Nsp4–ligand complexes [26, 27]. Six systems were generated and subjected to 100 ns MD simulations—one system estimated the stability of Nsp4 and the other five estimated the stability of the Nsp4–ligand complexes. The dynamic nature of the target protein and the docked-ligand complex was predicted in the presence of a solvent. All six systems were solvated in a box using a simple point-charge model. The topology of the ligands was created using ProDRG [28] and that for Nsp4 was generated using the GROMOS9653a6 force field [29]. The systems were neutralised by adding 1 Na^+^ ion. To eliminate steric hindrance, the steepest energy minimization was used for all systems in order to obtain the maximal force below 1000 kJ/mol/nm. Long-range electrostatic interactions were determined using the particle mesh Ewald (PME) method [30]. For computation of Lennard–Jones and Coulomb interactions, we used a radius cut-off of 1.0 nm; the LINCS algorithm was used to constrain H-bond lengths [31]. All simulations applied a consistent time step of 2 fs. Short-range non-bonded interactions were predicted using a 10-Å cut-off distance, whereas long-range electrostatics were predicted using the PME method with 1.6-Å Fourier grid spacing. Shake algorithms were used to fix all bonds, including H-bonds [32]. After energy minimisation, the systems were equilibrated, followed by position-restraint simulations under NVT and NPT conditions to maintain the volume, temperature, and pressure. Finally, all systems were subjected to a 100 ns MD simulation; coordinates were saved at 2 fs intervals. Root-mean-square deviation (RMSD), root-mean-square fluctuation (RMSF), radius of gyration (Rg), solvent-accessible surface area (SASA), H-bonds, and Gibbs free energy landscapes were calculated, and principal component analysis (PCA) was performed to predict correlated motions generated during protein–ligand interactions;‘gmx rms’, ‘gmx rmsf’, ‘gmx gyrate’, ‘gmx sasa’, ‘gmx hbond’, ‘gmx sham’, and ‘gmx covar’ (GROMACSv.2018.1; http://www.gromacs.org/), respectively, were used for these purposes. The resulting files were analysed and visualised using xmgrace (https://plasma-gate.weizmann.ac.il/Grace/).

### Binding-energy calculation

MM-PBSA is a widely used and well-accepted method for calculating the binding free energy of protein–ligand complexes [33]. Here, we used the g_mmpbsa tool (https://rashmikumari.github.io/g_mmpbsa/) to calculate the binding energy by integrating high-throughput MD simulation data [34]. The binding energy calculations can be described in the following equation:

$$ \Delta {\mathrm{G}}_{\mathrm{bind}}=\Delta {\mathrm{G}}_{\mathrm{mm}}+\Delta {\mathrm{G}}_{\mathrm{ps}}+\Delta {\mathrm{G}}_{\mathrm{nps}}-\mathrm{T}\Delta \mathrm{S} $$

Here, van der Waals and electrostatic interaction were calculated in molecular mechanics energy (ΔG_mm_). ΔG_ps_ and ΔG_nps_ are the polar and non-polar solvation energies, and TΔS is refer to the entropic contribution where temperature and entropy are denoted by T and S, respectively. The average binding energies of the top five protein–ligand complexes and amino acid residues contributing to the binding activity were calculated by using ‘python’ scripts included in the g_mmpbsa tool.

## Results

### Identification of lead compounds through structure-based virtual screening

Structure-based virtual screening enables the prediction of optimal interactions between ligands and a macromolecular target for complex formation. The ligands are subsequently sorted according to their binding free energy for the target. This requires the three-dimensional structure of the target, with the compounds obtained from a database and categorised according to their affinity. In the present study, we downloaded a subset of natural compounds (*n* = 97,999) from the ZINC database for virtual screening against PRRSV Nsp4. We subsequently identified the top 10 compounds sorted according to their minimum binding free energy (range:− 10.0 to − 9.2 kcal/mol) for further analyses (Table 1).

**Table 1:** Binding free energies of the top 10 screened compounds along with the amino acid residues involved in interactions. The amino acid residues shown in bold are involved in hydrogen-bonding interactions

S.No.	Compound (ZINC ID)	Binding free energy (Kcal/ mol)	Amino acid residues involved in interactions via different types of bonding
1.	ZINC38167083	−10.0	Ser18, Ala38, His39, Leu41, Thr42, Gly43, Asn44, Val61, **Gly63**, Asp64, Thr134, Ile143, Thr145, Phe151
2.	ZINC16919178	−9.9	Phe3, Thr5, Ser9, Leu10, **Asn11, **Phe26, Pro78, Tyr92, Leu94, Val99, Pro101, Ile123, Gly127
3.	ZINC08792350	−9.5	Phe3, Thr5, Ser9, Leu10, Asn11, Val76, Pro78, Arg90, Val99, Tyr92, Pro101, Phe166, Asp192, Ile123, Leu196
4.	ZINC01510656	−9.4	Thr5, Ser9, Leu10, Asn11, Phe26, Val76, Pro78, Tyr92, Leu94, Val99, IIe123, Gly127
5.	ZINC08877407	−9.3	**His39, **Gly63, Asp64, Ala114, Cys115, Gly116, ASP117, **Ser118, **His133, Thr134, Gly135, **Ser136, **Lys138, Ile143, Thr145, Phe151
6.	ZINC32124273	−9.3	Phe3, Thr5, Ser9, Asn11, Phe26, Pro78, Lys79, Ala80, Tyr92, Leu94, Arg90, Val99, Pro101, Ile123, Thr124, Glu125, Ala126, Gly127
7.	ZINC00852708	−9.2	Thr5, Ser9, Leu10, Asn11, Phe26, Val76, Pro78, Tyr92, Leu94, Val99, IIe123, Gly127
8.	ZINC01225926	−9.2	Thr5, Ser9, Leu10, Asn11, Phe26, Val76, Pro78, Leu94,Tyr92,Val99, Ile123, Gly127
9.	ZINC02116980	−9.2	Gly63, **Asp64, **Ala114, Cys115, **Gly116, ** Asp117,**Ser118, ** His133, Gly135, Thr134, **Ser136, **Lys138, ILE143, **Thr145, **Phe151
10.	ZINC08790125	−9.2	Ser18, Ala38, Leu41, Gly43, Asn44, His39, Val61, Gly63, Asp64, Thr134, IIe143, Thr145, Phe151

### Analysis and visualisation of the screened compounds

**Fig. 1:**
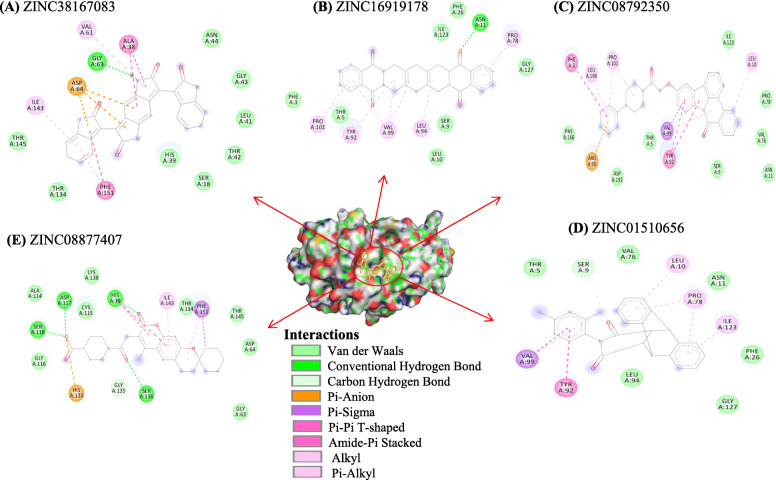
2D representation of the binding interactions of top five screened natural compounds with Nsp4 depicted key amino acid residues contributed in protein-ligand interactions. **A** ZINC38167083, (**B**) ZINC16919178, (**C**) ZINC08792350, (**D**) ZINC01510656, and (**E**) ZINC08877407

The active site residues of Nsp4 include His39, Asp64, and Ser118, as well as His133 and Ser136 that are reportedly essential for protein activity. The compound showing the optimal binding free energy (− 10.0 kcal/mol; ZINC38167083) demonstrated ligand interactions such as His39-mediated van der Waals interactions and Asp64-mediated Pi-anion interaction. Fig. 1 shows protein–ligand H-bond interactions involving the active site residues His39, Ser118, and Ser136, besides, Asp64 and His133 is interacted with van der Waals and pi-anion interaction with ZINC08877407. The interacting amino acid residues of top 10 compounds are shown in Table 1.

### Assessment of drug likeness through physicochemical property analysis

Physicochemical property analysis is one of the fundamental tasks in any drug discovery program. The top 10 screened compounds were then subjected to analysis of their physicochemical properties according to 7 principal descriptors (MW, logP value, status of HBDs and HBAs, 2D PSA, P, and VWSA). According to a previous study, a good drug should have an MW < 500 Da, an HBD < 5, and an HBA < 10. The MW, logP, HBD, and HBA of the selected compounds met the Lipinski’s rule. Additionally, PSA, P, and VWSA results displayed drug-like behaviour. (Table 2).

**Table 2:** Physicochemical properties of the top 10 screened compounds

S.No.	Compound	MW (g/mol)	LogP	HBD	HBA	PSA (2D) (Å)	P	VWSA (3D) (Å)
1.	ZINC38167083	446.422	3.317	4	4	116.40	45.91	494.66
2.	ZINC16919178	448.518	5.584	0	4	68.28	49.15	579.49
3.	ZINC08792350	488.547	4.32	0	6	67.67	57.27	660.92
4.	ZINC01510656	379.459	4.7	0	2	37.38	43.18	524.87
5.	ZINC08877407	453.535	3.995	0	6	93.14	47.17	667.66
6.	ZINC32124273	464.525	4.578	0	7	82.60	50.46	614.48
7.	ZINC00852708	365.432	4.392	0	2	37.38	41.41	492.81
8.	ZINC01225926	379.459	4.566	0	2	37.38	43.26	527.17
9.	ZINC02116980	477.488	4.828	0	6	97.05	50.01	641.83
10.	ZINC08790125	460.537	4.478	3	2	80.99	53.60	620.08

### MD simulation analysis

The structure of Nsp4 and top five screened compound-complex with Nsp4 was employed for 100 ns MD simulation study for predicting the dynamic changes during protein-ligand interaction and their nature of stability. The present study included various parameters i.e. RMSD, RMSF, Rg, SASA, H-bond, PCA, Gibbs free energy landscape, and binding free energy calculation.

#### Structural deviation analysis through RMSD

**Fig. 2:**
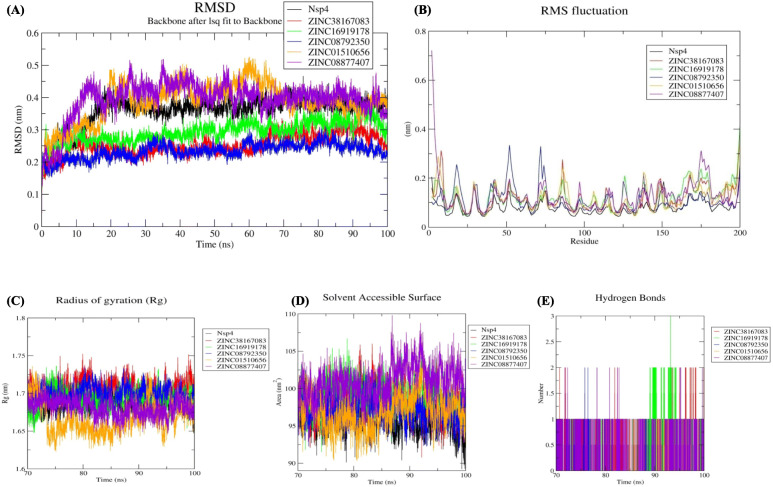
Stability analysis (**A**) RMSD values for the Nsp4–compound complexes. Flexibility analysis **(B)** RMSF values for the Nsp4–compound complexes over the final 30 ns of the simulations. Compactness (**C**) Rg, and Solvent accessible surface area analysis (**D**) SASA values for the final 30 ns of the simulations. Black, red, green, blue, orange, and violet colours represent Nsp4, Nsp4-ZINC38167083, Nsp4-ZINC16919178, Nsp4-ZINC08792350, Nsp4-ZINC01510656, and Nsp4-ZINC08877407, respectively. **E** Changes in the number of hydrogen bonds in each respective complex according to data from the final 30 ns of the simulations. Red, green, blue, orange, and violet colour represent Nsp4-ZINC38167083, Nsp4-ZINC16919178, Nsp4-ZINC08792350, Nsp4-ZINC01510656, and Nsp4-ZINC08877407 respectively

The RMSD value describes the dynamic behaviour among native structures to a new pose. After a 70 ns of simulation to obtain a stable trajectory, the RMSD values were 0.35, 0.25, 0.29, 0.23, 0.38, and 0.39 nm for Nsp4, Nsp4-ZINC38167083, Nsp4-ZINC16919178, Nsp4-ZINC08792350, Nsp4-ZINC01510656, and Nsp4-ZINC08877407, respectively. These data suggest that Nsp4-ZINC08792350 and Nsp4-ZINC38167083 are highly stable complexes relative to the others. Because each Nsp4–compound complex demonstrated stability after the 70 ns simulation, we performed further evaluations on each for last 30 ns trajectory (Fig. 2A).

#### Flexibility analysis through RMSF

Evaluation of the RMSF values used to assess structural rigidity revealed values of 0.08, 0.11, 0.12, 0.10, 0.11, and 0.11 nm for Nsp4, Nsp4-ZINC38167083, Nsp4-ZINC16919178, Nsp4-ZINC08792350, Nsp4-ZINC01510656, and Nsp4-ZINC08877407, respectively (Fig. 2B). Higher RMSF values were due to ligand binding, causing alterations in protein geometry. Minimal fluctuations were observed in Nsp4-ZINC08792350 and Nsp4-ZINC38167083 complex compared with that in other complexes.

#### Radius of gyration (Rg) analysis

Assessment of complex compactness according to Rg calculation revealed values of 1.50, 1.27, 1.43, 1.46, 1.39, and 1.44 nm for Nsp4, Nsp4-ZINC38167083, Nsp4-ZINC16919178, Nsp4-ZINC08792350, Nsp4-ZINC01510656, and Nsp4-ZINC08877407, respectively (Fig. 2C). The results indicate that the Nsp4-ZINC38167083 complex showed a more compact structure than the other complexes.

#### Solvent accessible surface area (SASA) analysis

To identify changes in the solvent-accessible regions of the complexes, we determined SASA values over the course of the final 30 ns of the simulation. Our study revealed values of 95.88, 98.33, 98.98, 97.13, 96.97, and 100.92 nm^2^ for Nsp4, Nsp4-ZINC38167083, Nsp4-ZINC16919178, Nsp4-ZINC08792350, Nsp4-ZINC01510656, and Nsp4-ZINC08877407 (Fig. 2D), revealing relatively minimal changes after binding by each of the compounds.

#### Interaction analysis through hydrogen bonding

Hydrogen bonding is the most important bond for stabilizing protein–ligand interactions. The average number of hydrogen bonds for the complexes Nsp4-ZINC38167083, Nsp4-ZINC16919178, Nsp4-ZINC08792350, Nsp4-ZINC01510656, and Nsp4-ZINC08877407 over the final 30 ns of the simulations was 0–1 and that for Nsp4-ZINC38167083 and Nsp4-ZINC16919178 was 0–2 and 0–3, respectively (Fig. 2E). Hence, these compounds interacted with Nsp4 and provided a stable complex during protein–ligand interactions.

#### Principal component analysis (PCA)

**Fig. 3:**
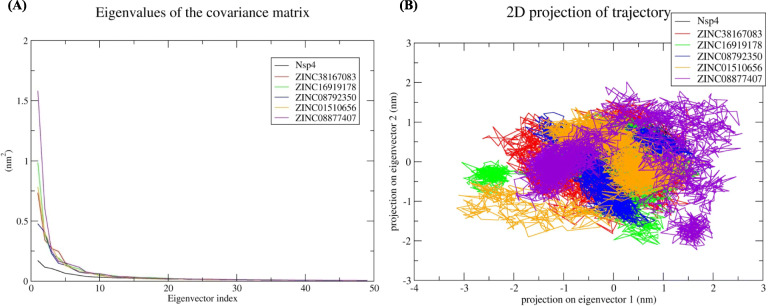
Principal component analysis **(A)** Eigenvalues derived from the final 30 ns of each simulation and used for PCA depicted Eigenvalues vs. first fifty eigenvector. **B** First two eigenvectors depicted Nsp4 motion in space for all the systems. Black, red, green, blue, orange, and violet colours represent Nsp4, Nsp4-ZINC38167083, Nsp4-ZINC16919178, Nsp4-ZINC08792350, Nsp4-ZINC01510656, and Nsp4-ZINC08877407 respectively

In PCA, the sum of the eigenvalues suggests the overall flexibility of a structure under different conditions. Therefore, the first 5 of 50 eigenvectors used to calculate eigenvalues from the final 30 ns of the simulation were used to determine the percentage change in structural movement. The results revealed that these five eigenvectors accounted for 42.85, 63.97, 63.27, 59.14, 64.83, and 71.05% of the motions for Nsp4, Nsp4-ZINC38167083, Nsp4-ZINC16919178, Nsp4-ZINC08792350, Nsp4-ZINC01510656, and Nsp4-ZINC08877407 respectively (Fig. 3A), suggesting increased movement after the binding of each ligand. Moreover, Nsp4-ZINC38167083, Nsp4-ZINC16919178, Nsp4-ZINC08792350, and Nsp4-ZINC01510656 showed less overall motion relative to Nsp4-ZINC08877407. Additionally, generation of a 2D plot for assessing protein dynamics after ligand binding suggested the overall stability (lowcorrelated motions) of Nsp4, Nsp4-ZINC38167083, and Nsp4-ZINC08792350(Fig. 3B), indicating these compounds as possible leads for further evaluation as inhibitors.

#### Gibbs free energy landscape

**Fig. 4:**
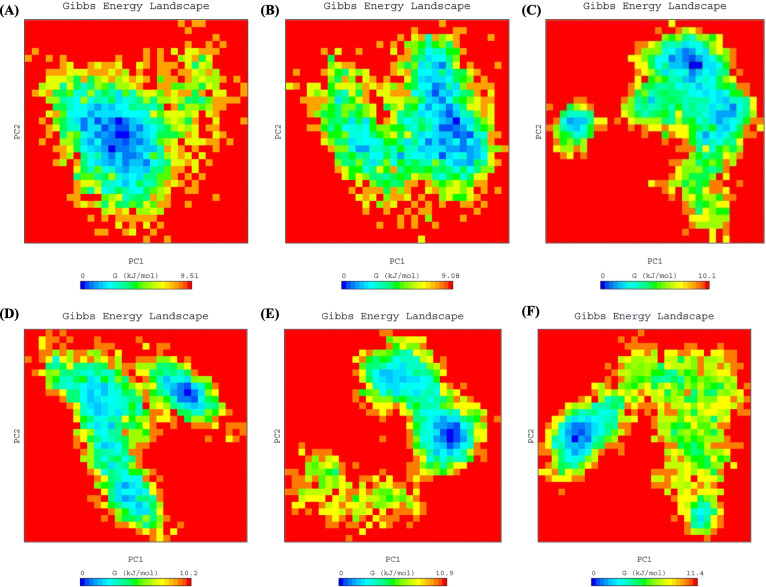
The color-coded illustration of the Gibbs free energy landscape plotted using PC1 and PC2. The lower energy systems are represented by the deeper blue color on the contour map. **A** Nsp4, (**B**) Nsp4-ZINC38167083, (**C**) Nsp4-ZINC16919178, (**D**) Nsp4-ZINC08792350, (**E**) Nsp4-ZINC01510656, and (**F**) Nsp4-ZINC08877407

We then calculated the Gibbs free energy landscape using the first two principal components (PC1 and PC2) in order to visualize the results. Fig. 4 shows the colour-coded plots generated for Nsp4 along with each complex. The lowest free energy values (≤9.08 kJ/mol) were observed for Nsp4-ZINC38167083, suggesting that this complex demonstrated overall thermodynamic stability. The other complexes (Nsp4-ZINC16919178, Nsp4-ZINC08792350, Nsp4-ZINC01510656, and Nsp4-ZINC08877407) had values of to 11.4 kJ/mol, implying that these complexes have numerous high-energy minima.

### Binding free energy

We then evaluate the binding free energy associated with each ligand through MM-PBSA using the final 10 ns of the simulation, for calculation of van der Waals and electrostatic interactions, Polar solvation, and SASA. The calculated binding free energy for Nsp4-ZINC38167083, Nsp4-ZINC16919178, Nsp4-ZINC08792350, Nsp4-ZINC01510656, and Nsp4-ZINC08877407 was − 124.54, − 128.44, − 159.33, − 122.50, and − 78.19 kJ mol^− 1^ respectively (Table 3).

**Table 3:** Average binding free energies of Nsp4 complexes in kJ mol ^− 1^

Compounds	van der Waals interactions	Electrostatic interactions	Polar solvation	SASA	Binding energy
ZINC38167083	−161.742 ± 16.571	−36.716 ± 14.192	89.570 ± 30.038	−15.652 ± 1.890	−124.540 ± 17.142
ZINC16919178	−202.964 ± 19.700	−14.995 ± 12.321	107.288 ± 24.499	−17.775 ± 1.752	−128.446 ± 13.116
ZINC08792350	− 210.397 ± 12.126	−5.732 ± 4.973	76.102 ± 13.673	−19.303 ± 1.248	−159.330 ± 14.200
ZINC01510656	−145.554 ± 11.730	−3.108 ± 4.634	40.772 ± 10.125	−14.615 ± 1.279	−122.505 ± 12.199
ZINC08877407	−101.513 ± 9.428	−7.238 ± 5.481	40.488 ± 23.110	−9.935 ± 2.168	−78.199 ± 21.645

**Fig. 5:**
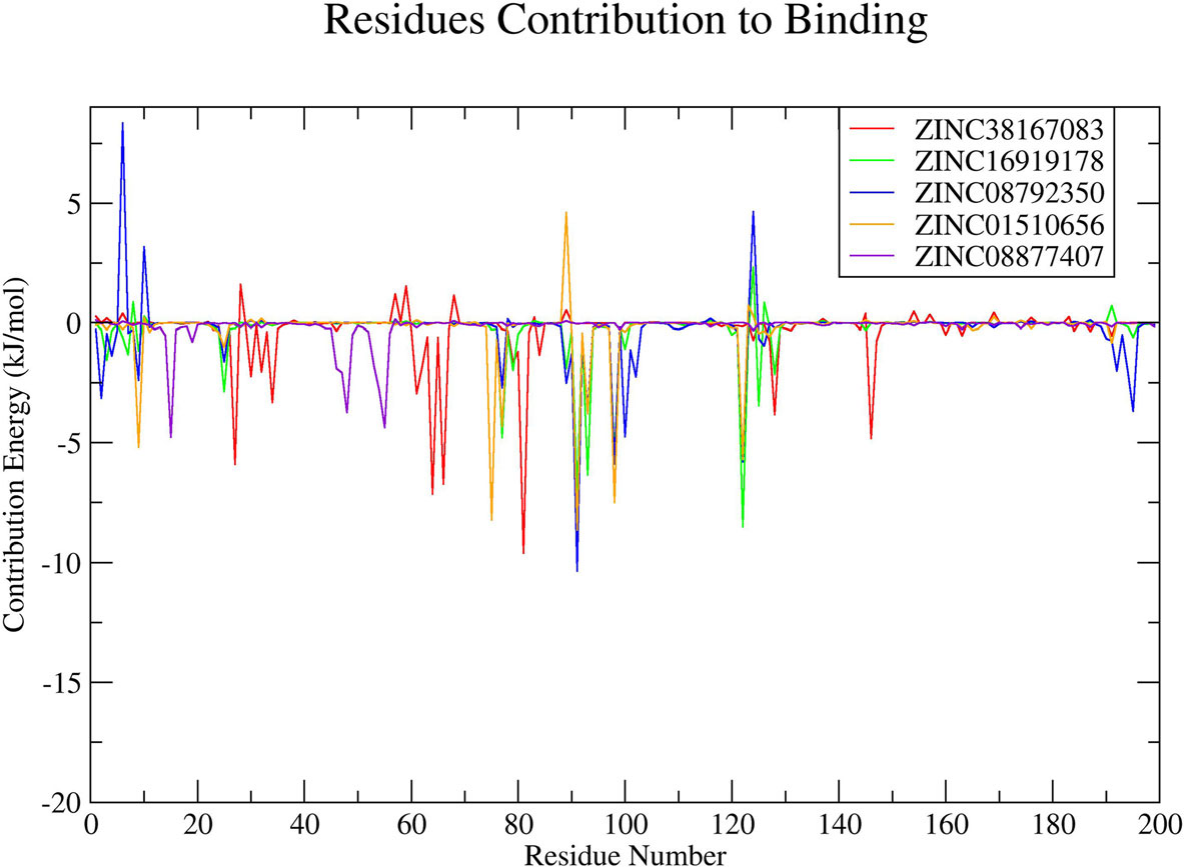
Plot depicting the amino acid residues of Nsp4 contributing to the binding with natural compounds. Red, green, blue, orange, and violet colours represent ZINC38167083, ZINC16919178, ZINC08792350, ZINC01510656, and ZINC08877407, respectively

The investigation of residual binding energy is a key method for identifying residues important to ligand binding. Fig. 5 shows that amino acid residues at positions 5 to 142 contributed significantly to binding of ZINC38167083, ZINC16919178, ZINC08792350, and ZINC01510656, which are the catalytic residues in the active site. Fewer contacts were observed in relation to ZINC08877407 binding, suggesting that ZINC38167083, ZINC16919178, ZINC08792350, and ZINC01510656 represent potential Nsp4 inhibitors.

## Discussion

PRRSV is a recalcitrant and intricate disease in a pig when working as a cofactor in a porcine respiratory disease complex (PRDC) or primary infectious agent. It was identified as the most frequent virus linked to PRDC [35–39]. Furthermore, PRRSV has been shown to impair the host immune system, which can lead to more serious secondary infections, and chronic disorders [35]. The involvement of Nsp4 in PRRSV replication and pathogenesis is decoded and recommended as one of the key molecular targets for drug development [14]. Therefore, identification of Nsp4 inhibitors is needed to prevent and manage the disease. Natural compounds have made immense contributions in the identification of lead molecule(s) with antiviral potential. It is believed that the disease can be controlled successfully by developing small molecules that can inhibit Nsp4 activity linked with pathogenesis [14]. In the present study, computational approaches are utilized for the identification of possible lead compounds via molecular docking of natural compounds database through structure-based virtual screening followed by downstream analysis. Structure based-virtual screening is a powerful computational approach that is used to investigate important lead molecule(s) from a big set of a compound database based on the lowest binding energy required for stabilizing the protein-ligand complex [40].

From the structure-based virtual screening, we have selected the top ten natural compounds that show interaction with key residues. Further, protein-ligand analysis of the top 5 compounds demonstrated that the ZINC38167083 interacted with Nsp4 and formed one conventional hydrogen bond at position Gly63. Besides, amino acid residues Ser18, His39, Leu41, Thr42, Gly43, Asn44, Thr134, and Thr145 were involved in van der Waals interactions, and Val61 and Ile143 formed alkyl bonds. Ala38 and Phe151 participated in interaction through amide-pi and pi-pi t-shaped bonding. Additionally, Asp64 contributed to interaction through the pi-anion bond. ZINC16919178 bonded with Nsp4 at position Asn11 by one conventional hydrogen bond. In addition, amino acid residues Phe3, Thr5, Ser9, Leu10, Phe26, Ile123, and Gly127 formed van der Waals interactions; Pro78, Tyr92, Leu94, Val99, and Pro101 formed alkyl and pi-alkyl bonds. ZINC08792350 interacted with Nsp4 Thr5, Ser9, Asn11, Val76, Pro78, Ile123, Phe166, and Asp192 through van der Waals interactions; Phe3 and Tyr92 formed pi-pi t-shaped bonding. In addition, the amino acid residues Leu10, Pro101, Leu196 contributed to interaction through pi-alkyl bonding; Arg90 and Val99 formed pi-anion and pi-sigma bonds with ZINC08792350, respectively. ZINC01510656 bonded with Nsp4 at Thr5, Asn11, Val76, Phe26, Leu94, and Gly127 through van der Waals interactions; amino acid residues Leu10, Pro78, and IIe123 formed pi-alkyl bonds, and Ser9, Tyr92, Val99 contributed in interaction by carbon-hydrogen bonding, pi-pi t-shaped, and pi-sigma interactions, respectively. ZINC08877407 formed conventional hydrogen bonds with Nsp4 at position His39, Ser118, ASP117, and Ser136; amino acid residues Gly63, Asp64, Ala114, Cys115, Gly116, Thr134, Lys138, and Thr145 contributed to protein-ligand interaction through van der Waals. Additionally, Gly135 formed a carbon-hydrogen bond, and His133, Ile143, and Phe151 interacted through pi-anion, pi-alkyl, and pi-sigma bonding, respectively. Medicinal chemists have traditionally been interested in noncovalent interactions that are indicative of attraction, directed intermolecular forces in their search for the “glue” that keeps ligand and their molecular target together. In recent years, with the rapid increase in the number of solved biomolecular structures and the performance enhancement of computational methods, it is now possible to provide a more thorough understanding of protein-ligand interaction [41]. Therefore, based on the results, it was concluded that the screened compounds can inhibit the virulence activity of Nsp4 [14]. Besides, the results of physicochemical properties prediction suggest that the screened compounds demonstrated good drug-like behavior and could be considered for further analysis [42, 43]. Therefore, 100 ns MD simulation analysis was conducted for Nsp4 and top 5 natural compounds i.e. ZINC38167083, ZINC16919178, ZINC08792350, ZINC01510656, and ZINC08877407, respectively with Nsp4 to evaluate the dynamic behavior of protein and protein-ligand complexes. It is recognized as a powerful approach for predicting the conformational stability of macromolecules before and after ligand binding, besides the simulated data can be utilized for calculation of real binding energy of small molecules concerning time along with a contribution of binding amino acid residues present in the macromolecular target [44]. Several structural parameters were calculated, including RMSD, RMSF, Rg, SASA, H-bonding, PCA, and gibbs free energy [45–48]. The RMSD value indicated that all of the complexes were stable and creating an equilibrated trajectory for further investigation. As a result, we determined RMSF, Rg, SASA, PCA, and Gibbs free energy to determine the nature of each system subjected for MD simulation. Drug selectivity, metabolization, and stability all require H-bonds. To better understand the H-bond and its contributions to the overall stability of each system, an H-bond analysis of natural compounds-Nsp4 complexes were calculated. The hydrogen bonding study indicates that all of the Nsp4-complexes are stable and made bonding with essential catalytic residues [49]. The overall analysis revealed that each complex was stabilizing after 70 ns indicating better interaction with Nsp4 in terms of stability that is required for its inhibition. Further, MM-PBSA binding free energy and residual binding energy were calculated to assess the binding affinities of natural compounds with Nsp4. For determining the binding free energy of protein–ligand complexes by using MD simulation trajectory, it is a frequently used and well-accepted method [33, 50]. The strength of the binding contacts between the ligand and the target protein is measured by ligand binding affinity, which is directly linked to ligand potency. In the field of drug discovery, its evaluation is crucial. Furthermore, in favorable reactions, the free energy is negative. So, lowering the binding energy improves interactions, and low binding energy corresponds to the high binding affinity of protein-ligand complexes [51]. MM-PBSA analysis demonstrated that the compounds ZINC38167083, ZINC16919178, ZINC08792350, and ZINC01510656 can act as a potential lead for inhibition of Nsp4 [52, 53]. Whereas, ZINC08877407 was not recommended as a lead because their binding energy was found to be higher as compared to other compounds.

In past years, the identification of lead compounds for drug development take much time and cost as well as required good infrastructure experimental facilities [11, 54]. Due to advances in structural biology, computer science, and bioinformatics, it becomes easy to find out putative molecule(s) by a screening of a big database that has a strong affinity with the target for experimental evaluation [24, 55]. It saves the cost and time of the scientific community. Most of the medicines available in the market are from a natural source or it is a derivative of naturally occurring molecules [11, 24]. Natural compounds have immense potential to inhibit virus and pathogenic proteins and act as antiviral drugs [56–58]. The results presented in this work are, therefore, informative for understanding the antiviral potential of suggested compounds as therapeutics for PRRSV. It might be also useful for the prevention of pigs and other animals from different viral diseases [59, 60].

## Conclusions

PRRSV infection is a main concern for the global swine industry, and there is a need to identify novel and effective therapeutic agents. Given the importance of Nsp4 in PRRSV replication and pathogenesis, we employed computational and MD approaches to screen and identify natural compounds as novel inhibitors of Nsp4 activity. The results identified four possible lead compounds that represent potentially effective drug-like inhibitors for application as antiviral therapeutics. Further studies are warranted to confirm these findings through experimental and clinical evaluations in order to promote future management of PRRSV infection.

## Data Availability

All data generated or analysed during this study are included in the manuscript.

## Abbreviations

2D: Two-dimensional
3CLSP: 3C-like serine protease
HBA: Hydrogen-bond acceptor
HBD: Hydrogen-bond donor
MD: Molecular dynamics
MM-PBSA: Molecular mechanics Poisson–Boltzmann surface area
MW: Molecular weight
Nsp4: Non-structural protein 4
ORF: Open reading frame
PL1/2pro: Papain-like protease
PME: Particle mesh Ewald
PRRSV: Porcine reproductive and respiratory syndrome virus
PSA: Polar surface area
VWSA: van der Waals surface area

## References

[1] Huang C, Bernard D, Zhu J, Dash RC, Chu A, Knupp A, Hakey A, Hadden MK, Garmendia A, Tang Y (2020). Small molecules block the interaction between porcine reproductive and respiratory syndrome virus and CD163 receptor and the infection of pig cells. Virol J.

[2] Cavanagh D (1997). Nidovirales: a new order comprising Coronaviridae and Arteriviridae. Arch Virol.

[3] Benfield DA, Nelson E, Collins JE, Harris L, Goyal SM, Robison D, Christianson WT, Morrison RB, Gorcyca D, Chladek D (1992). Characterization of swine infertility and respiratory syndrome (SIRS) virus (isolate ATCC VR-2332). J Vet Diagn Investig.

[4] Music N, Gagnon CA (2010). The role of porcine reproductive and respiratory syndrome (PRRS) virus structural and non-structural proteins in virus pathogenesis. Anim Health Res Rev.

[5] Meng XJ (2000). Heterogeneity of porcine reproductive and respiratory syndrome virus: implications for current vaccine efficacy and future vaccine development. Vet Microbiol.

[6] Sun N, Li E, Wang Z, Zhao J, Wang S, He J, Bai Y, Li H (2014). Sodium tanshinoneIIA sulfonate inhibits porcine reproductive and respiratory syndrome virus via suppressing N gene expression and blocking virus-induced apoptosis. Antivir Ther.

[7] Zhou DH, Wang X, Yang M, Shi X, Huang W, Feng Q (2013). Combination of low concentration of (−)-epigallocatechin gallate (EGCG) and curcumin strongly suppresses the growth of non-small cell lung cancer in vitro and in vivo through causing cell cycle arrest. Int J Mol Sci.

[8] Du T, Nan Y, Xiao S, Zhao Q, Zhou EM (2017). Antiviral strategies against PRRSV infection. Trends Microbiol.

[9] Evans AB, Dong P, Loyd H, Zhang J, Kraus GA, Carpenter S (2017). Identification and characterization of small molecule inhibitors of porcine reproductive and respiratory syndrome virus. Antivir Res.

[10] Zitterl-Eglseer K, Marschik T (2020). Antiviral medicinal plants of veterinary importance: a literature review. Planta Med.

[11] Agnihotry S, Pathak RK, Srivastav A, Shukla PK, Gautam B (2020). Molecular docking and structure-based drug design. Computer-aided drug design.

[12] Singh DB, Pathak RK (2020). Computational approaches in drug designing and their applications. Experimental protocols in biotechnology.

[13] Ziebuhr J, Snijder EJ, Gorbalenya AE (2000). Virus-encoded proteinases and proteolytic processing in the Nidovirales. Microbiol.

[14] Tian X, Lu G, Gao F, Peng H, Feng Y, Ma G, Bartlam M, Tian K, Yan J, Hilgenfeld R, Gao GF (2009). Structure and cleavage specificity of the chymotrypsin-like serine protease (3CLSP/nsp4) of porcine reproductive and respiratory syndrome virus (PRRSV). J Mol Biol.

[15] Shi Y, Lei Y, Ye G, Sun L, Fang L, Xiao S, Fu ZF, Yin P, Song Y, Peng G (2018). Identification of two antiviral inhibitors targeting 3C-like serine/3C-like protease of porcine reproductive and respiratory syndrome virus and porcine epidemic diarrhea virus. Vet Microbiol.

[16] An TQ, Li JN, Su CM, Yoo D (2020). Molecular and cellular mechanisms for PRRSV pathogenesis and host response to infection. Virus Res.

[17] Irwin JJ, Sterling T, Mysinger MM, Bolstad ES, Coleman RG (2012). ZINC: a free tool to discover chemistry for biology. J Chem Inf Model.

[18] Dalby A, Nourse JG, Hounshell WD, Gushurst AK, Grier DL, Leland BA, Laufer J (1992). Description of several chemical structure file formats used by computer programs developed at molecular design limited. J Chem Inf Comput Sci.

[19] O'Boyle NM, Banck M, James CA, Morley C, Vandermeersch T, Hutchison GR (2011). Open babel: an open chemical toolbox. J Chem.

[20] Trott O, Olson AJ (2010). AutoDock Vina: improving the speed and accuracy of docking with a new scoring function, efficient optimization, and multithreading. J Comput Chem.

[21] Berman HM, Westbrook J, Feng Z, Gilliland G, Bhat TN, Weissig H, Shindyalov IN, Bourne PE (2000). The protein data bank. Nucleic Acids Res.

[22] Pettersen EF, Goddard TD, Huang CC, Couch GS, Greenblatt DM, Meng EC, Ferrin TE (2004). UCSF chimera—a visualization system for exploratory research and analysis. J Comput Chem.

[23] Goodsell DS, Morris GM, Olson AJ (1996). Automated docking of flexible ligands: applications of AutoDock. J Mol Recognit.

[24] Pathak RK, Singh DB, Sagar M, Baunthiyal M, Kumar A (2020). Computational approaches in drug discovery and design. Computer-aided drug design.

[25] Jejurikar BL, Rohane SH (2021). Drug designing in discovery studio. Asian J Res Chem.

[26] Pronk S, Páll S, Schulz R, Larsson P, Bjelkmar P, Apostolov R, Shirts MR, Smith JC, Kasson PM, Van Der Spoel D, Hess B (2013). GROMACS 4.5: a high-throughput and highly parallel open source molecular simulation toolkit. Bioinformatics.

[27] Abraham MJ, Murtola T, Schulz R, Páll S, Smith JC, Hess B, Lindahl E (2015). GROMACS: high performance molecular simulations through multi-level parallelism from laptops to supercomputers. SoftwareX.

[28] Schüttelkopf AW, Van Aalten DM (2004). PRODRG: a tool for high-throughput crystallography of protein–ligand complexes. Acta Crystallogr D Biol Crystallogr.

[29] Oostenbrink C, Villa A, Mark AE, Van Gunsteren WF (2004). A biomolecular force field based on the free enthalpy of hydration and solvation: the GROMOS force-field parameter sets 53A5 and 53A6. J Comput Chem.

[30] Darden T, York D, Pedersen L (1993). Particle mesh Ewald: an N· log (N) method for Ewald sums in large systems. J Chem Phys.

[31] Hess B, Bekker H, Berendsen HJ, Fraaije JG (1997). LINCS: a linear constraint solver for molecular simulations. J Comput Chem.

[32] Ryckaert JP, Ciccotti G, Berendsen HJ (1977). Numerical integration of the cartesian equations of motion of a system with constraints: molecular dynamics of n-alkanes. J Comput Phys.

[33] Genheden S, Ryde U (2015). The MM/PBSA and MM/GBSA methods to estimate ligand-binding affinities. Expert Opin Drug Discovery.

[34] Kumari R, Kumar R (2014). Open source drug discovery consortium and Lynn, A. g_mmpbsa-a GROMACS tool for high-throughput MM-PBSA calculations. J Chem Inf Model.

[35] Lim B, Kim S, Lim KS, Jeong CG, Kim SC, Lee SM, Park CK, Te Pas MF, Gho H, Kim TH, Lee KT (2020). Integrated time-serial transcriptome networks reveal common innate and tissue-specific adaptive immune responses to PRRSV infection. Vet Res.

[36] Brockmeier SL, Palmer MV, Bolin SR (2000). Effects of intranasal inoculation of porcine reproductive and respiratory syndrome virus Bordetella bronchiseptica, or a combination of both organisms in pigs. Am J Vet Res.

[37] Allan G, McNeilly F, Kennedy S, Meehan B, Ellis J, Krakowka S (2000). Immunostimulation, PCV-2 [porcine circovirus] and PMWS [porcine wasting syndrome]. Vet Rec.

[38] Thacker EL, Halbur PG, Ross RF, Thanawongnuwech R, Thacker BJ (1999). Mycoplasma hyopneumoniae potentiation of porcine reproductive and respiratory syndrome virus-induced pneumonia. J Clin Microbiol.

[39] Brockmeier SL, Halbur PG, Thacker EL, Brogden KA, Guthmiller JM (2002). Porcine respiratory disease complex. Polymicrobial diseases. Chapter 13.

[40] Maia EHB, Assis LC, de Oliveira TA, da Silva AM, Taranto AG (2020). Structure-based virtual screening: from classical to artificial intelligence. Front Chem.

[41] Zhou P, Huang J, Tian F (2012). Specific noncovalent interactions at protein-ligand interface: implications for rational drug design. Curr Med Chem.

[42] Lipinski CA, Lombardo F, Dominy BW, Feeney PJ (1997). Experimental and computational approaches to estimate solubility and permeability in drug discovery and development settings. Adv Drug Deliv Rev.

[43] Jiao Y, Peng J, Ye X, Hu H, Gan L, Yang J, et al. Study on pharmacological properties and cell absorption metabolism of novel daidzein napsylates. Royal Society open. 2021;science 8(1):201475. 10.1098/rsos.201475 PMC7890489 33614082

[44] Shukla R, Singh TR (2021). High-throughput screening of natural compounds and inhibition of a major therapeutic target HsGSK-3 β for Alzheimer’s disease using computational approaches. J Genet Eng Biotechnol.

[45] Pathak RK, Gupta A, Shukla R, Baunthiyal M (2018). Identification of new drug-like compounds from millets as xanthine oxidoreductase inhibitors for treatment of hyperuricemia: a molecular docking and simulation study. Comput Biol Chem.

[46] Rai SK, Pathak RK, Singh DB, Bhatt A, Baunthiyal M (2021). Chemo-informatics guided study of natural inhibitors targeting rho GTPase: a lead for treatment of glaucoma. In Silico Pharm.

[47] Shukla R, Singh TR. Identification of small molecules against cyclin dependent kinase-5 using chemoinformatics approach for Alzheimer’s disease and other tauopathies. J Biomol Struct Dyn. 2020:1–13. 10.1080/07391102.2020.1844050. 10.1080/07391102.2020.1844050 33155527

[48] Karthick V, Nagasundaram N, Doss CGP, Chakraborty C, Siva R, Lu A, Zhang G, Zhu H (2016). Virtual screening of the inhibitors targeting at the viral protein 40 of Ebola virus. Infect Dis Pov.

[49] Agrawal S, Govind Kumar V, Gundampati RK, Moradi M, Kumar TK (2021). Characterization of the structural forces governing the reversibility of the thermal unfolding of the human acidic fibroblast growth factor. Sci Rep.

[50] Kumar VG, Agrawal S, Kumar TK, Moradi M. Binding affinity estimation from restrained umbrella sampling simulations. bioRxiv. 2021. 10.1101/2021.10.28.466324. 10.1038/s43588-022-00389-9 PMC10766565 38177953

[51] Wan S, Bhati AP, Zasada SJ, Coveney PV (2020). Rapid, accurate, precise and reproducible ligand–protein binding free energy prediction. Interface Focus.

[52] Poli G, Granchi C, Rizzolio F, Tuccinardi T (2020). Application of MM-PBSA methods in virtual screening. Molecules.

[53] Kuhn B, Gerber P, Schulz-Gasch T, Stahl M (2005). Validation and use of the MM-PBSA approach for drug discovery. J Med Chem.

[54] Eisenstein M (2020). Active machine learning helps drug hunters tackle biology. Nat Biotechnol.

[55] Schenone M, Dančík V, Wagner BK, Clemons PA (2013). Target identification and mechanism of action in chemical biology and drug discovery. Nat Chem Biol.

[56] Omrani M, Keshavarz M, Nejad Ebrahimi S, Mehrabi M, McGaw LJ, Ali Abdalla M, Mehrbod P (2020). Potential natural products against respiratory viruses: a perspective to develop anti-COVID-19 medicines. Front Pharmacol.

[57] Martinez JP, Sasse F, Brönstrup M, Diez J, Meyerhans A (2015). Antiviral drug discovery: broad-spectrum drugs from nature. Nat Prod Rep.

[58] Bhattacharya R, Dev K, Sourirajan A (2021). Antiviral activity of bioactive phytocompounds against coronavirus: an update. J Virol Methods.

[59] Li WG, Dai FY, Cheng YX, Yin GF, Bi JL, Li DP (2013). Identification of porcine reproductive and respiratory syndrome virus inhibitors through an oriented screening on natural products. Chem Res Chin Univ.

[60] Gómez-García M, Puente H, Argüello H, Mencía-Ares Ó, Rubio P, Carvajal A. In vitro assessment of antiviral effect of natural compounds on porcine epidemic diarrhea coronavirus. Front Vet Sci. 2021;8. 10.3389/fvets.2021.652000. 10.3389/fvets.2021.652000 PMC8039285 33855058

